# Fast stomatal kinetics in sorghum enable tight coordination with photosynthetic responses to dynamic light intensity and safeguard high water use efficiency

**DOI:** 10.1093/jxb/erae389

**Published:** 2024-09-18

**Authors:** Martin W Battle, Silvere Vialet-Chabrand, Piotr Kasznicki, Andrew J Simkin, Tracy Lawson

**Affiliations:** School of Life Sciences, University of Essex, Wivenhoe Park, Colchester CO4 3SQ, UK; School of Life Sciences, University of Essex, Wivenhoe Park, Colchester CO4 3SQ, UK; School of Life Sciences, University of Essex, Wivenhoe Park, Colchester CO4 3SQ, UK; School of Life Sciences, University of Essex, Wivenhoe Park, Colchester CO4 3SQ, UK; School of Life Sciences, University of Essex, Wivenhoe Park, Colchester CO4 3SQ, UK; MPI of Molecular Plant Physiology, Germany

**Keywords:** Dynamic light, photosynthesis, photosynthetic induction, *Sorghum bicolor*, speed of stomata, stomatal anatomy, stomatal conductance, water use efficiency

## Abstract

In this study, we assessed 43 accessions of sorghum from 16 countries across three continents. Our objective was to identify stomatal and photosynthetic traits that could be exploited in breeding programmes to increase photosynthesis without increasing water use under dynamic light environments. Under field conditions, sorghum crops often have limited water availability and are exposed to rapidly fluctuating light intensities, which influences both photosynthesis and stomatal behaviour. Our results highlight a tight coupling between photosynthetic rate (*A*) and stomatal conductance (*g*_s_) even under dynamic light conditions that results in steady intrinsic water use efficiency (*W*_i_). This was mainly due to rapid stomatal responses, with the majority of sorghum accessions responding within ≤5 min. The maintenance of the ratio of the concentration of CO_2_ inside the leaf (*C*_i_) and the surrounding atmospheric concentration (*C*_a_) over a large range of accessions suggests high stomatal sensitivity to changes in *C*_i_, that could underlie the rapid *g*_s_ responses and extremely close relationship between *A* and *g*_s_ under both dynamic and steady-state conditions. Therefore, sorghum represents a prime candidate for uncovering the elusive mechanisms that coordinate *A* and *g*_s_, and such information could be used to design crops with high *A* without incurring significant water losses and eroding *W*_i_.

## Teaser

Sorghum accession have high water use efficiency due to rapid stomatal behaviour and synchrony between *A* and *g*_s_ .

## Introduction

Improvements to crop varieties via breeding, along with improved farming practices, have led to the yields of major crops increasing dramatically since the start of the green revolution of the 1960s (Pingali, 2012; Ort and Long, 2014). The majority of these increases were brought about by advances in agricultural practices and the introduction of dwarf varieties that reduced lodging, enhanced light capture, and improved harvest index [a measure of the ratio of harvestable produce/yield (e.g. grain) relative to the crop biomass] (Ray *et al.*, 2013; Long *et al.*, 2015). Breeding practices largely assumed that crops can be grown under ideal conditions, and therefore water requirements and nutrient uptake to date have not been the focus of breeding efforts, and, for this reason, few improvements have been made (Pingali, 2012; Ort and Long, 2014). As such, alongside the near-tripling of crop yield, there has been a comparable increase in agricultural water demand (Pingali, 2012; Ort and Long, 2014). Currently, >80% of the world’s fresh water is used in agriculture (WWAP, 2015; D’Odorico *et al.*, 2020) and, as fresh water becomes less readily available due to diminishing groundwater supplies (Dalin *et al.*, 2017), this percentage could be pushed even higher (Dai, 2013; Karmakar *et al.*, 2017; Spinoni *et al.*, 2018). Furthermore, the predicted rise in global temperatures by as much as 2 °C will lead to more sporadic rainfall worldwide, with a declining frequency of precipitation predicted (Dai, 2013; Karmakar *et al.*, 2017; Spinoni *et al.*, 2018; IPCC, 2022), therefore water availability presents one of the main challenges for agriculture in the near future (Leakey *et al.*, 2019). Enhancing the photosynthetic performance of current crop varieties without an increase in water use, as well as increasing the utilization of less common crop species, are pivotal steps toward harnessing available water resources more efficiently. These advancements will be instrumental in achieving the necessary crop productivity to meet global demand (Flexas, 2016; FAO *et al*., 2018; Kayatz *et al.*, 2019; Leakey *et al.*, 2019).

Crops with C_4_ photosynthesis possess a carbon-concentrating mechanism (CCM) maintaining high CO_2_ concentration near the carboxylation sites of Rubisco, improving the efficiency of photosynthesis and lowering transpiration, especially in high temperature conditions when evaporative demand is high (Pearcy and Ehleringer, 1984). The CCM mechanism tends to reduce diffusional limitations by stomata and decreases water use (Pearcy and Ehleringer, 1984; Way *et al.*, 2014) compared with C_3_ crops, highlighting their potential use in future agriculture. Intrinsic water use efficiency (*W*_i_), defined as the photosynthetic rate (*A*) divided by stomatal conductance to water vapour (*g*_s_), describes the biological control of the balance between CO_2_ uptake and water loss, and is generally higher in C_4_ compared with C_3_ crops (Blum, 2009; Lawson *et al.*, 2010; Lawson and Blatt, 2014). *W*_i_ is determined by leaf anatomy (e.g. stomatal size, density, and aperture) and biochemistry (e.g. photosynthesis capacity) and has been selected for in breeding programmes in the past for improved crop yield (Condon *et al.*, 2002, 2004).

In natural environments, changes in the angle of the sun, cloud cover, and shading from overlapping leaves or neighbouring plants mean that plants are continually exposed to rapid changes in light intensities and spectral properties that have major consequences for photosynthetic carbon assimilation (Pearcy, 1990; Chazdon and Pearcy, 1991; Pearcy and Way, 2012). Photosynthetic photon flux density (PPFD)-driven changes in *A* occur within several tens of seconds to minutes, compared with changes in *g*_s_ which can take up to tens of minutes to reach a new steady state (Barradas and Jones, 1996; Lawson and Morison, 2004; Lawson *et al.*, 2010; Vico *et al.*, 2011; McAusland *et al.*, 2016). A slow increase in *g*_s_ can limit *A*, whilst a slow decrease results in a lag between the drop in *A* and *g*_s_ response. The delay between *A* and *g*_s_ can result in additional water loss for a reduced carbon gain, lowering *W*_i_ (Hetherington and Woodward, 2003; Franks and Farquhar, 2007; Brodribb *et al.*, 2009; Lawson *et al.*, 2010, 2012; Vico *et al.*, 2011; Drake *et al.*, 2013; McAusland *et al.*, 2016). As *W*_i_ is determined by both *A* and *g*_s_, it is important to consider the impact that both parameters can have on *W*_i_. Although high *W*_i_ is desirable, this should not be at the expense of *A*, and therefore *g*_**s**_ rates that are more in line with mesophyll demands for CO_2_ should promote higher *A* and maintain *W*_i_; however, this is only true if stomatal responses to changing conditions are not too slow (McAusland *et al.*, 2016; Lawson and Vialet-Chabrand, 2019). Although species specific, sluggish stomatal responses limit photosynthesis by ~10% (McAusland *et al.*, 2016) and reduce *W*_i_ by 20% (Lawson and Blatt, 2014; De Souza *et al.*, 2020; Acevedo-Siaca *et al.*, 2021). Increasing the speed of stomatal responses has therefore become a key target for improved *A* and *W*_i_ (Lawson and Blatt, 2014; Qu *et al.*, 2016; Lawson and Vialet-Chabrand, 2019; Papanatsiou *et al.*, 2019). The significant variation known to exist in stomatal kinetics (Vico *et al.*, 2011; Lawson *et al.*, 2012; Drake *et al.*, 2013; McAusland *et al.*, 2016; Faralli *et al.*, 2019b; Eyland *et al.*, 2021) could be used to identify the underlying genetic variation that influences the speed of stomatal responses, an unexploited target for improving photosynthesis and/or *W*_i_ (Faralli *et al.*, 2019b). Qu *et al.* (2020), for example, applied a genome-wide association study (GWAS) and successfully identified a candidate gene (an Na^+^/H^+^ tonoplastic antiporter OsNHX1) that regulated the speed of stomatal closure.

Sorghum (*Sorghum bicolor* L. Moench) is the second most important C_4_ crop globally (USDA, 2022), grown as a traditional, rain-fed, staple grain crop in semi-arid regions with high air and soil surface temperatures and low soil quality, environments in which C_3_ crops struggle to thrive (Rao *et al.*, 2006). Recently, variations in anatomical features such as leaf width and stomatal density have been shown to drive differences in *W*_i_ in a range of sorghum accessions (Pan *et al.*, 2022; Al-Salman *et al.*, 2023). In these studies, a strong correlation between *A* and *g*_s_ was reported that in most C_3_ plant species disappears under fluctuating light environments (Lawson *et al.*, 2012; McAusland *et al.*, 2016; Faralli *et al.*, 2019a, b). The mechanism coordinating *A* and *g*_s_ in C_3_ plants is elusive and is thought to depend on a positive feedback loop controlling stomatal aperture based on mesophyll photosynthetic signal (Lawson *et al.*, 2014, 2018), with *C*_i_ having long been proposed as the signal coordinating *A* and *g*_s_ (Farquhar *et al.*, 1978; Vialet-Chabrand *et al.*, 2021). Furthermore, many studies have reported that changes in stomatal aperture result in a constant ratio between the concentration of CO_2_ inside the leaf (*C*_i_) and the surrounding atmospheric concentration (*C*_a_) (*C*_i_:*C*_a_ ratio; Ball *et al*., 1987; Mott, 1988). This is an appealing prospect as *C*_i_ is determined by mesophyll consumption of CO_2_ and the flux of CO_2_ into the leaf, which is controlled by stomatal conductance (Lawson *et al.*, 2018). However, several studies have suggested that stomatal responses to *C*_i_ are insufficient to account for the changes observed in *g*_s_ in response to light (Raschke, 1975; Sharkey and Raschke 1981; Farquhar and Sharkey, 1982). More recent studies have also demonstrated *g*_s_ responses to light when *C*_i_ was held constant (Messinger *et al.*, 2006; Lawson *et al*., 2008), suggesting that *C*_i_ cannot be the only signal. However, most of these investigations have been performed on C_3_ plants, and the mechanism coordinating this relationship may be different in C_4_ plants due to the CCM leading to different stomatal behaviour under fluctuating light conditions.

In this study, we assessed natural variation in stomatal and photosynthetic traits and *W*_i_ in 43 accessions of sorghum from 16 countries across three continents (Supplementary Table S1). Using this information, one of our main questions was: what is the the dynamic coordination between *A* and *g*_s_ and how does this impact *W*_i_ under fluctuating light intensity in a C_4_ crop such as sorghum? C_4_ crops possess a lower maximum *g*_s_ due to their lower stomatal size and density (Taylor *et al.*, 2012) which potentially influences their rapidity of response (McAusland *et al.*, 2016).

## Materials and methods

### Plant material used in this study

Seed material for the 43 accessions of sorghum (*Sorghum bicolor*) used in this study were kindly provided by Dr Alison Bently (NIAB, Cambridge), Dr Dagmar Janovská (Crop Research Institute, Prague), and Dr Jana Kholova and Dr Sunita Choudhary (ICRISAT, Hyderabad). A full list of accessions used can be found in Supplementary Table S1. Further information on IS lines can be found at www.genesys-pgr.org and on EC lines in Brahmi *et al.* (2015). These accessions include: (i) a range of traditional cultivars or landraces which have been grown as traditional crops in their native region; (ii) advanced or improved cultivars that are the product of modern breeding programmes; and (iii) breeding and research material with notable traits which make them useful parental lines for breeding programmes or notable outliers for research (https://glis.fao.org/glis/).

### Growth conditions

Sorghum seed were initially sown on damp tissue paper and stored in the dark at 26 °C for 5 d to induce germination. Healthy seedlings were then transplanted to pots containing peat-based compost (Levingtons F2S, Everris, Ipswich, UK). Plants were grown under controlled conditions in a controlled-environment room, with a constant temperature of 26 °C. Humidity was maintained above 60% using a Trotec B400 humidifier (Trotec, Heinsberg, Germany). Plants were grown under a 10 h:14 h light:dark cycle with daytime lighting maintained at 1000 µmol m^–2^ s^–1^ of full-spectrum white light provided by either LightDNA-8 (Valoya, Helsinki, Finland) or DYNA (Heliospectra, Gothenburg, Sweden) LED grow-lights. Plants were grown in individual pots that were randomly distributed and rotated every 2 d. Plants were watered as required to maintain sufficient soil moisture, with supplemental nutrients provided with weekly application of Hoagland’s solution (Hoagland and Arnon, 1950). Before testing, plants were grown to the fifth-leaf stage of growth, which typically took 3–5 weeks. All measurements were taken on the youngest fully expanded primary leaf at the time of testing.

### Infra-red gas exchange measurements of photosynthesis

Gas exchange parameters were measured on the youngest fully expanded leaf using a Li-6400 or Li-6800 infrared gas analyser (Li-Cor, Lincoln, NE, USA). For all gas exchange experiments in this study, the internal CO_2_ concentration of the leaf cuvette (*C*_a_) was set to 400 μmol mol^−1^, leaf temperature to 26 °C, and relative air humidity within the cuvette to 60%.

When measuring the response of *A* to PPFD (*A*/*Q* response curves), leaves were initially acclimated under a light irradiance above saturation (2000 mmol m^–2^ s^–1^) until net photosynthetic CO_2_ assimilation (*A*) had stabilized, at which point the first data point was recorded. PPFD was then decreased in 12 steps (2000, 1500, 1250, 1000, 750, 500, 300, 200, 100, 50, 20, and 0 mmol m^–2^ s^–1^), with a new recording being taken at each new light level once *A* had reached a new steady state (typically 1–3 min per step). Data were fitted to a rectangular hyperbola plot of the Michaelis–Menten equation in Rstudio (RStudioTeam, 2020) as previously described in Stevens *et al.* (2021). The initial slope of these modelled curves was used to calculate the maximum apparent quantum yield of net carbon assimilation (QY) for each accession; these values were then compared using a one-way ANOVA using the aov function in Rstudio. Statistically significant differences (*P*≤0.05) were then grouped using a Tukey’s post-hoc test.

The response to a rapid increase in PPFD (step change response to light) was measured by first allowing *A* to stabilize at a PPFD of 100 mmol m^–2^ s^–1^. Once a steady state was reached, measurements were taken every 10 s for 5 min in order to determine baseline parameters at low light. PPFD was then increased to 1000 mmol m^–2^ s^–1^ and further readings were taken every 10 s for 30 min. Data were fitted in Rstudio (RStudioTeam, 2020), using the model presented in McAusland *et al.* (2016). The time constant for the assimilation rate (τ_ai_) and stomatal opening (τ_i_) were calculated using the excel macro provided in Vialet-Chabrand *et al.* (2017c). A one-way ANOVA was used to assess differences between accessions and, where a significant difference was found, a Tukey’s post-hoc test was used to pool accessions into statistically separable groupings.

### Measurement of stomatal anatomical characteristics

After gas exchange measurements were completed, stomatal impressions were taken from the abaxial and adaxial leaf surfaces of the same leaf using Xantopren L blue silicone impression material and hardener (Beyer Dental, Leverkusen, Germany) utilizing the method described in Weyers and Johansen (1985). Impression material was allowed to dry fully before being removed from the leaf, after which clear nail varnish was applied to the impression in order to produce a positive replica, which was removed using clear sticky tape and applied to a microscope slide. Light microscopy images were taken using an SC500 5MP microscope digital camera (Swift Optical Instruments, Texas, USA) fitted to a Leica ATC 2000 compound microscope (Leica Microsystems, Wetzlar, Germany) at ×10 objective magnification. The ×10 magnification images were used to take stomatal density measurements in ImageJ (Schneider *et al.*, 2012).

A separate group of plants from 10 selected accessions (EC884904, IS1054, IS10876, IS14556, IS16044, IS24953, IS29472, IS2950, IS23496, and IS36556) were grown and additional impressions were taken. For these plants, in addition to stomatal density, guard cell length and stomatal pore length measurements were made using the tools in the SC500 5MP microscope digital camera’s accompanying software, Swift Imaging 3.0 (Swift Optical Instruments). Measurements were made on live images at ×20 objective magnification and exported to Microsoft Excel for further analysis. Maximum anatomical stomatal conductance (anatomical *g*_smax_) was calculated from these characteristics using the equation by Dow *et al*. (2014):


$$\begin{array}{l} Anatomical\;g_{smax}=\frac{\left(d\times SD\times a_{max}\right)}{\left(v\times \left(\text{l}+\left(\frac{\pi }{2}\right)\times \sqrt{\left(\frac{a_{max}}{\pi }\right)}\right)\right)} \end{array}$$

Where *d* is the diffusivity of water in air (24.6 × 10^6^ m^2^ s^–1^ at 25 °C; Dow and Bergmann, 2014) and *v* is the molecular volume of air (24.4 × 10^3^ m^3^ mol^–1^ at 25 °C and 101.3 kPa (Dow and Bergmann, 2014). *a*_max_, the maximal stomatal pore area (µm^2^), was calculated from the measured pore length, assuming that stomatal pores are elliptical, with a major axis equal to the pore length and the minor axis estimated at half the pore length (McElwain *et al.*, 2016). Stomatal pore depth, *l* (µm) was estimated as a quarter of the guard cell length.

Statistical analyses of stomatal characteristics were performed in Rstudio (RStudioTeam, 2020). A one-way or two-way ANOVA was used to analyse single-factor or two-factor differences, respectively. Where a significant difference was identified, a Tukey’s post-hoc test was performed to group factors by significance.

## Results

### Photosynthetic and stomatal responses to light intensity

**Fig. 1.**
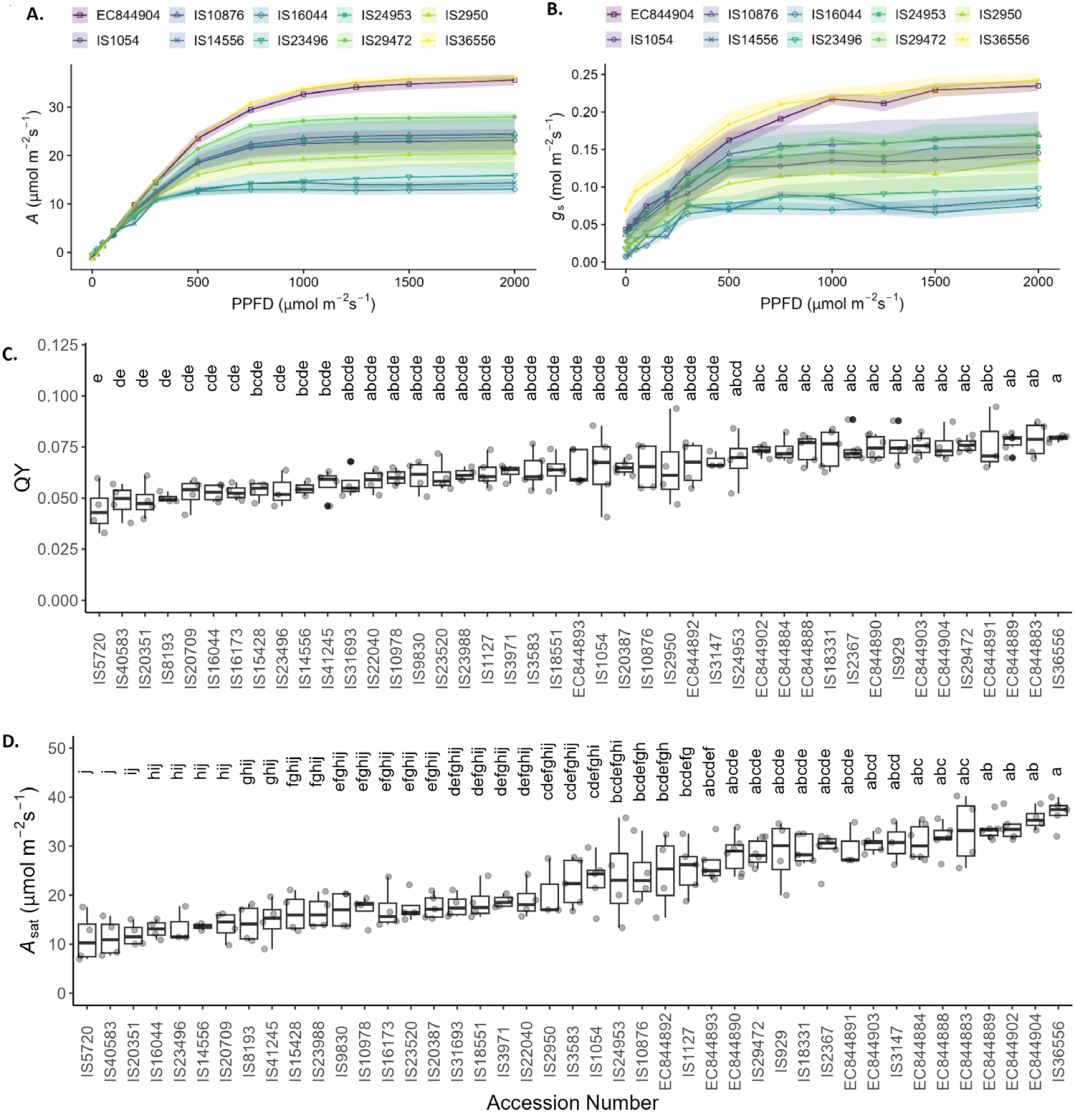
The effect of light on photosynthetic and stomatal parameters. Light responses of sorghum plants showing (A) CO_2_ assimilation rate (*A*) and (B) stomatal conductance to water (*g*_s_) in 10 representative accessions under a range of PPFD irradiance intensities from 0 to 2000 µmol m^–2^ s^–1^. (C) Maximum apparent quantum yield of net carbon assimilation (QY) for 43 sorghum accessions calculated from the initial slope of the *A*/PPFD curves for each accession. Accession numbers in (C) are ordered from lowest to highest mean QY. (D) Photosynthetic CO_2_ assimilation at 2000 µmol m^–2^ s^–1^ (*A*_sat_) of 43 sorghum accessions. Accession numbers are ordered from lowest to highest mean *A*_sat_. In (C) and (D), colours indicate the biological status of the accession as shown in Supplementary Table S1, and letters indicate significantly different groupings of ANOVA results (*P*≤0.05) calculated using a Tukey’s test. *n*=3–8. The shaded ribbon represents the SE around the mean.

To identify differences in the CO_2_ assimilation rates (*A*; Fig. 1A) and stomatal conductance to water vapour (*g*_s_; Fig. 1B) between the 43 tested accessions (Supplementary Fig. S1A, B), we examined *A* and *g*_s_ as a function of light intensity. Ten examples shown in Fig. 1A and B illustrate the resulting light response curves for *A* and *g*_s_, selected to represent the full range of responses observed. As expected, *A* initially increased linearly with increasing light intensity before saturating and plateauing at intensities between 500 µmol m^–2^ s^–1^ and 800 µmol m^–2^ s^–1^. Interestingly *g*_s_ showed a similar response pattern to that described for *A*. As light intensity was changed every 2–3 min, these experiments also provide information on the speed of the dynamic responses of both *A* and *g*_s_ to changing light intensity. As a result of the similar patterns of *A* and *g*_s_, the *C*_i_:*C*_a_ ratio in all accessions was maintained fairly constant (~ 0.25–0.35) at all light intensities apart from those below 250 µmol m^–2^ s^–1^ (Supplementary Fig. S1C).

The initial slope of the light response curves was used to calculate the maximum apparent quantum yield of CO_2_ assimilation (QY; Fig. 1C) whilst the plateau provided a measure of the light-saturated rate of CO_2_ assimilation (*A*_sat_; Fig. 1D) (and photosynthetic capacity) for each sorghum accession. The QYs of the 43 tested accessions were variable and can broadly be placed into five, heavily overlapping statistical groups (a–e) (Fig. 1C). The accession with the highest average maximum apparent quantum yield, IS36556, a traditional cultivar from Nigeria, had a significantly higher apparent quantum yield than the 12 lines with the lowest QYs. It should be noted that no individual cultivar observed stood alone as statistical anomaly, with all being statistically similar to at least 27 other cultivars, which was also the case for the accession with the lowest average QY, IS5720 (a traditional cultivar from India). *A*_sat_ also revealed large differences between sorghum accessions (Fig. 1D). These data split our accessions into 10 statistical groups (a–j), with IS36556 and IS5720 also having the highest and lowest *A*_sat_, respectively. When combining data for all accessions, *A*_sat_ correlated strongly with maximum apparent quantum yield (*R*=0.94, *P*<2.2e-16; Supplementary Fig. S1D).

**Fig. 2.**
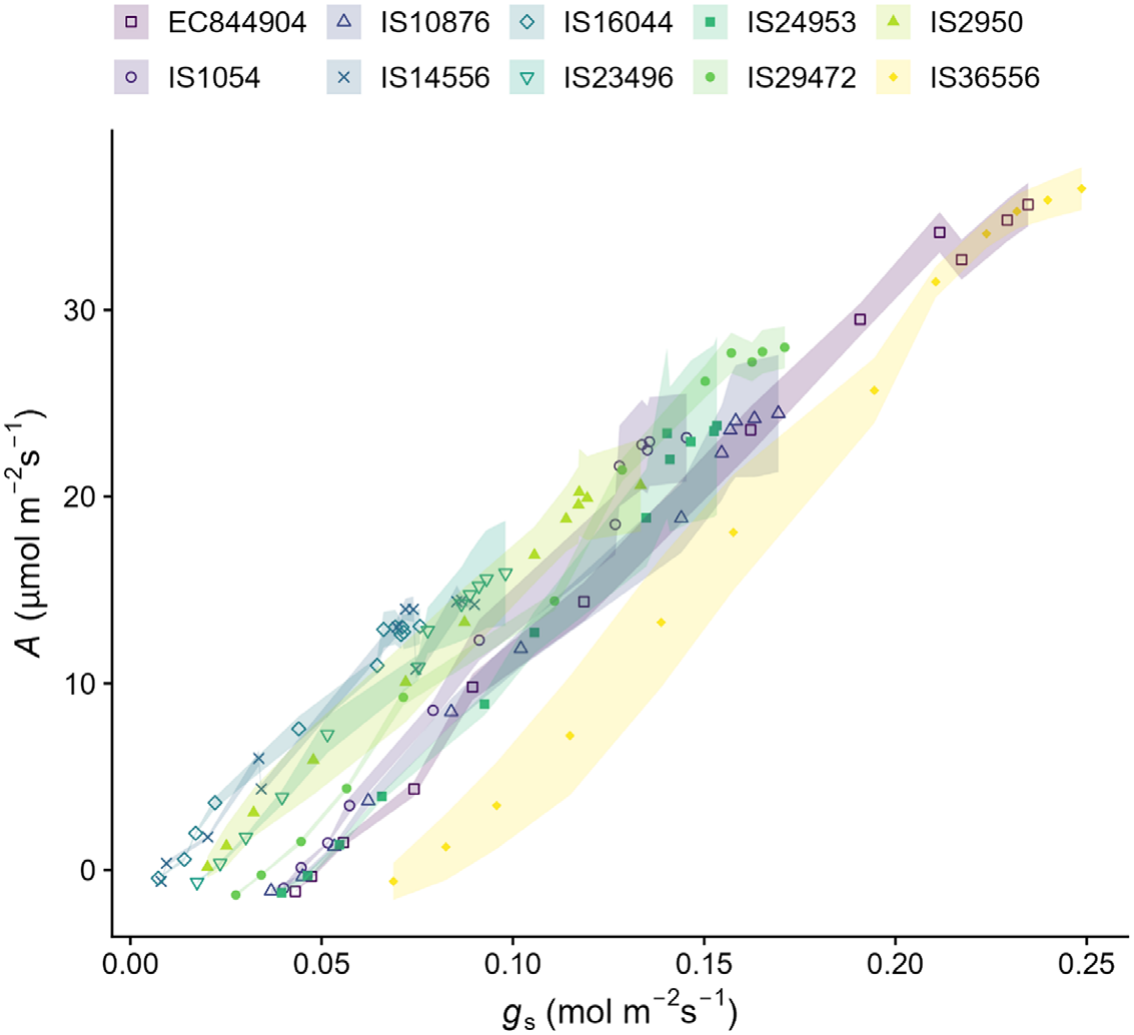
Rate of change of CO_2_ assimilation rate (*A*) relative to stomatal conductance to water (*g*_s_) in response to changes in PPFD irradiance intensities from 0 to 2000 µmol m^–2^ s^–1^. Data are shown from 10 representative accessions. Points indicate the mean average of 4–8 plants, and ribbons indicate the SE.

**Fig. 3.**
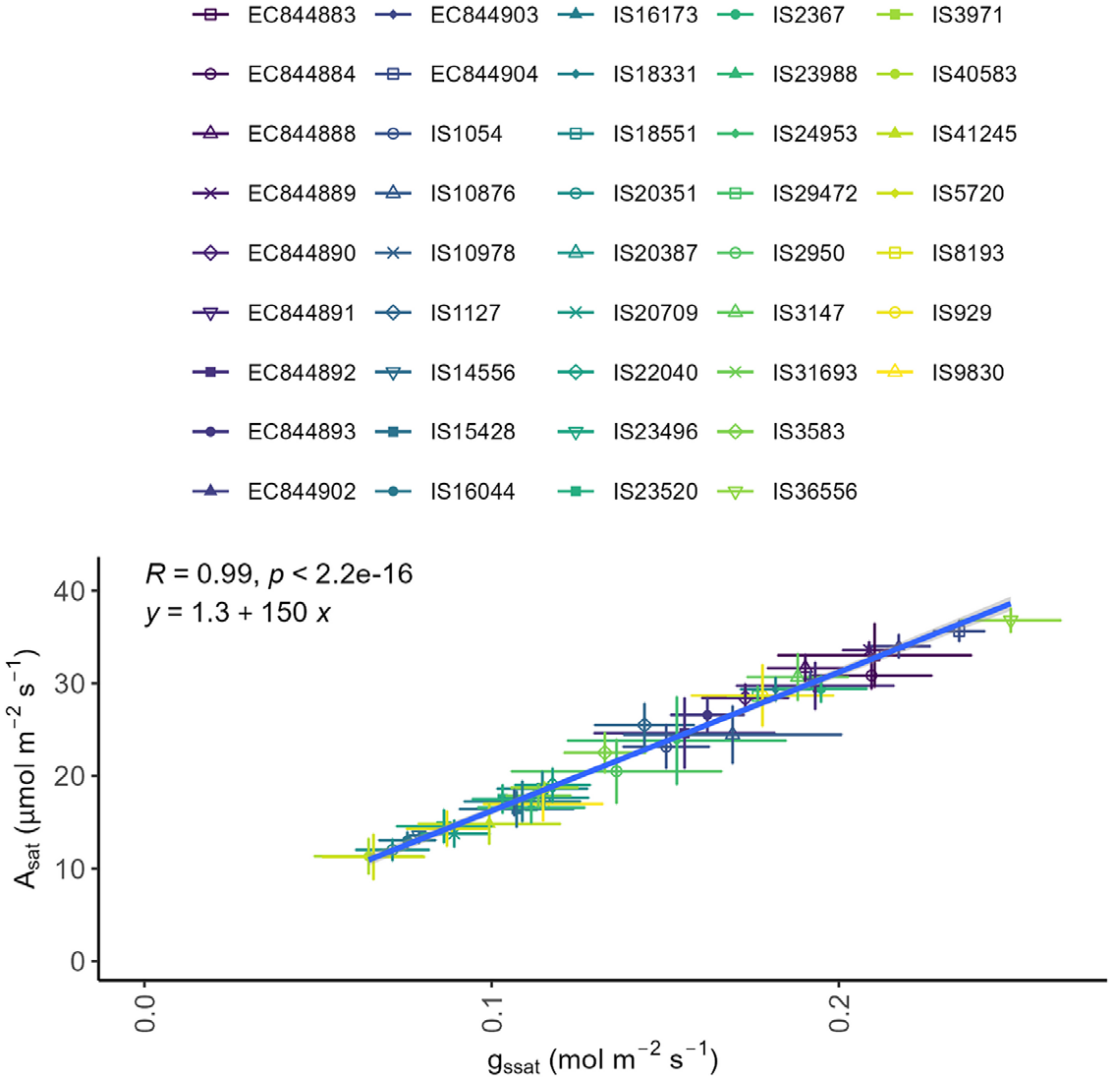
Light-saturated rate of photosynthetic CO_2_ assimilation (*A*_sat_; *A* at 2000 µmol m^–2^ s^–1^) relative to stomatal conductance under saturating light (*g*_ssat_; *g*_s_ at 2000 µmol m^–2^ s^–1^) for each tested sorghum accession. Points indicate mean average values for accessions as indicated by colour. Error bars indicate the SE of the mean, *n*=3–8. The line represents *R*=0.99, *P*<2.2e-16 with a regression formula of *y*=1.3 + 150*x*.

A strong positive correlation (regression coefficient for all tested accessions *R*>0.96, *P*-values <5.9e-07) was observed between *g*_s_ and *A* at all irradiances from 0 to 2000 µmol m^–2^ s^–1^ (Fig. 2; Supplementary Fig. S2A). It should be noted that a saturation of *A* can be seen in some accessions at maximal *g*_s_. In the 10 selected accessions, the regression formulae of these relationships range from IS16044 (*y*= –1.7 + 200*x*) to IS36556 (*y*= –17 + 220*x*), illustrating near identical slopes for these 10 accessions. While, typically, the linearity of this correlation indicates a relatively stable *W*_i_, the variation in *g*_s_ at low *A* values suggests differences in *W*_i_ between accessions (Fig. 2). It is also apparent from these relationships that the cultivars with the lowest *g*_s_ values tend to have lower light-saturated rates of photosynthesis (Fig. 2). For example, IS4556 generally has a higher *W*_i_ than EC844904, at the same *A* (due to lower *g*_s_ values). However, at saturating light, IS4556 *A*_sat_ is significantly lower (~15µmol m^–2^ s^–1^) than that of EC844904 (~35 µmol m^–2^ s^–1^). Plotting *A*_sat_ against *g*_s_ under saturating light intensities (*g*_ssat_; calculated from the plateau of the data shown in Fig. 1B) for each accession (Fig. 3) also reveals a ver*y* strong correlation between these parameters across all 43 accessions (*R*=0.99, *P*<2.2e-16).

### Speed of responses to a step increase in irradiance varies between Sorghum accessions

**Fig. 4.**
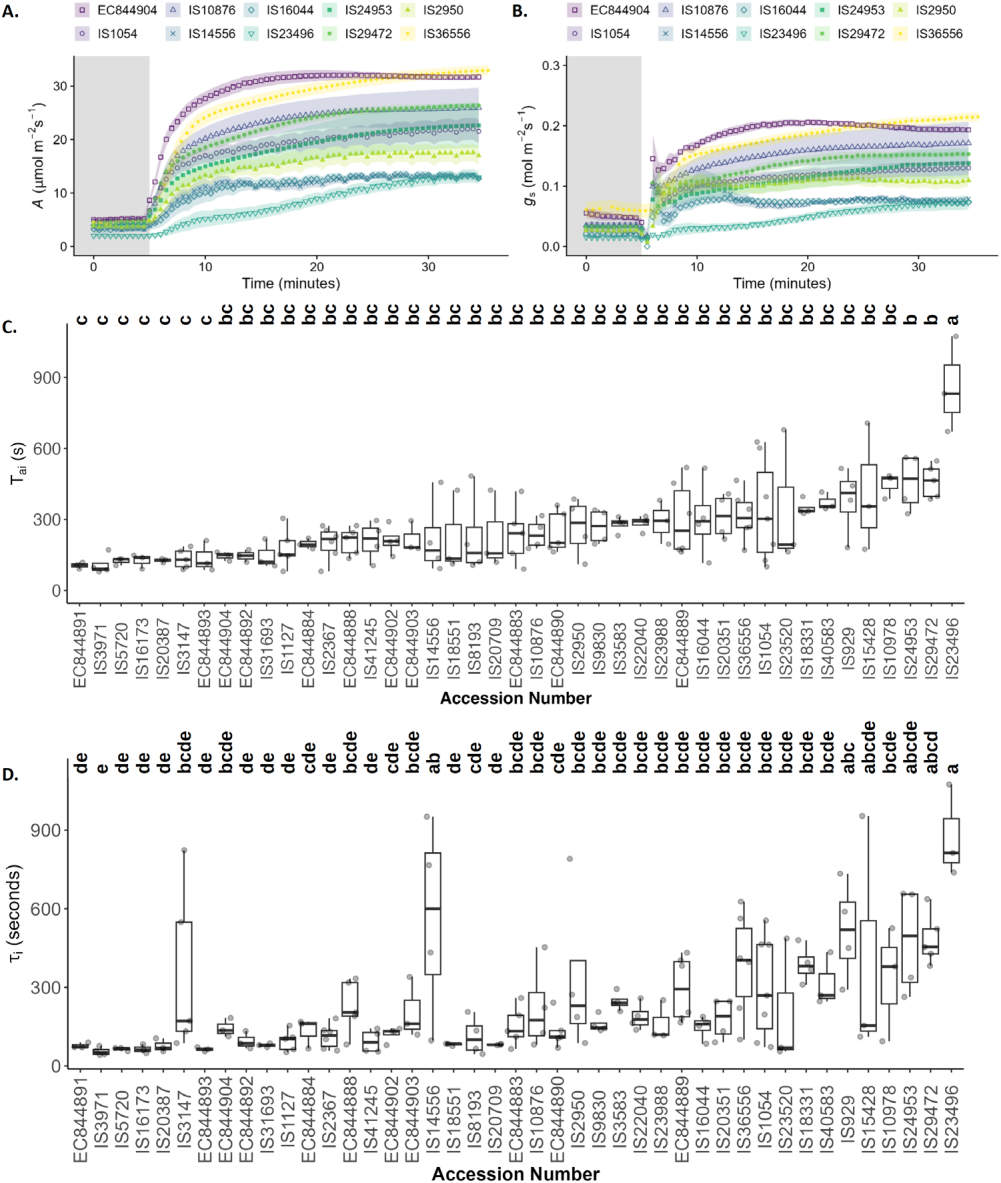
Stomatal and photosynetic kinetic responses. Temporal responses to a rapid increase in PPFD irradiance from 100 µmol m^–2^ s^–1^ (shaded area) to 1000 µmol m^–2^ s^–1^ showing changes in (A) CO_2_ assimilation rate (*A*) and (B) stomatal conductance to water (*g*_s_) in 10 representative sorghum accessions. Data from this protocol were used to calculated time constants of the (C) light-saturated rate of carbon assimilation (τ_ai_) and (D) stomatal opening (τ_i_) in seconds, shown here for each tested sorghum accession. Accession numbers in (C) and (D) are ordered from the lowest to highest mean τ_ai_. In (C) and (D), colours indicate the biological status of the accession as shown in Supplementary Table S1, and letters indicate significantly different groupings of ANOVA results (*P*≤0.05) calculated using a Tukey’s test. The shaded ribbon represent the SE around the mean.

Sorghum plants exposed to a step increase in light intensity from 100 µmol m^–2^ s^–1^ to 1000 µmol m^–2^ s^–1^ showed a typical hyperbolic response in both *A* (Fig. 4A) and *g*_s_ (Fig. 4B). However, significant variation was observed in the final *A* and *g*_s_ values between the different cultivars, with values ranging from 32.95 µmol m^–2^ s^–1^ (IS36556) to 12.74 µmol m^–2^ s^–1^ (IS16044) for *A*, and 0.21 mol m^–2^ s^–1^ (IS36556) to 0.07 mol m^–2^ s^–1^ (IS23496) for *g*_s_. Visualizing the responses in the 10 selected lines, it is clear that the responses of *A* to changing irradiance were similar to the *g*_s_ responses for each accession. This is further illustrated in the relatively stable *C*_i_ values in the majority of the 10 accessions observed during this kinetic response (Supplementary Fig. S3).

In order to parameterize these response kinetics, we used the model presented in McAusland *et al.* (2016) from which time constants for *A* (τ_ai_; Fig. 4C; Supplementary Fig. S4A) and *g*_s_ (τ_i_; Fig. 4D; Supplementary Fig. S4B) were determined for each of our accessions. Values for τ_ai_ (Fig. 4C) ranged from 92 s to 1235 s and could be separated into two statistically significant groups (a and b), while τ_i_ (Fig. 4D) ranged from 42 s to 1062 s and fitted into three significant but overlapping groups (a–c). IS23496, a traditional cultivar from Ethiopia, was the only accession observed to fit into group ‘a’ for τ_ai_ and τ_i_, with a significantly slower response for both *A* and *g*_sw_ to the step increase in irradiance compared with all other cultivars examined. While τ_ai_ and τ_i_ did not always align on an accession-by-accession basis (Supplementary Fig. S4), when τ_ai_ and τ_i_ for all cultivars were plotted against each other (Supplementary Fig. S5), a strong linear correlation was observed (*R*=0.61, *P*<0.05). Despite the synchronicity of the rapidity of response, τ_ai_ and τ_i_ were not correlated to steady-state *g*_s_ and *A* values. It is noteworthy that parameters describing the initial and final *g*_s_ and *A* values during photosynthesis induction were significantly intercorrelated (*P*<0.05), with low initial values leading to low final values.

### Intrinsic water use efficiency under steady-state irradiance

**Fig. 5.**
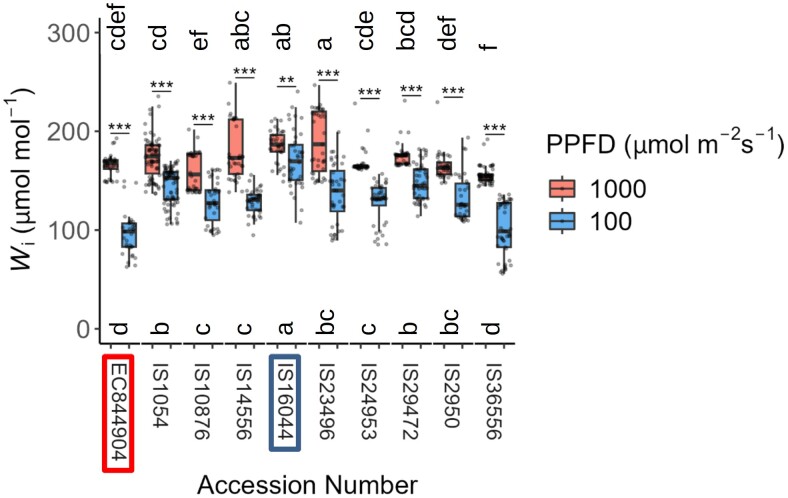
Changes in intrinsic water use efficiency (*W*_i_) in response to PPFD irradiance. Average *W*_i_ measured every 30 s for 5 min of dark-adapted plants exposed to 100 µmol m^–2^ s^–1^ of light (blue boxplots) compared with average *W*_i_ measured every 30 s for 5 min after 30 min of exposure to 1000 µmol m^–2^ s^–1^ of PPFD irradiance (red boxplots). The graph shows data from 10 representative sorghum accessions. Asterisks (*) above accessions indicate a significant difference between plants treated with 100 µmol m^–2^ s^–1^ and 1000 µmol m^–2^ s^–1^ for the given accession calculated by ANOVA (***P<0.0001, **P=0.001–0.01, *P=0.01–0.05). Letters indicate significantly different groupings of ANOVA results (*P*≤0.05) of accessions for plants exposed to 100 µmol m^–2^ s^–1^ (letters below the boxplots) or 1000 µmol m^–2^ s^–1^ (letters above the boxplots) of PPFD irradiance. Groupings were calculated using a post-hoc Tukey’s test, *n*=4–8. Highlighted accession numbers identify the lowest *g*_smax_ (red box) and the second lowest *g*_smax_ (blue box).

The kinetic responses of *W*_i_ (*A*/*g*_s_) were determined from the data collected from the step increase in irradiance (see Fig. 4). As the kinetic responses were relatively stable over time (see Supplementary Fig. S6) and differences were only really apparent at 100 µmol m^–2^ s^–1^ and 1000 µmol m^–2^ s^–1^ PPFD, steady-state *W*_i_ was determined from the last 5 min at each light intensity (Fig. 5; Supplementary Fig. S7A).

*W*_i_ varies greatly between the 10 selected accessions at both 100 µmol m^–2^ s^–1^ and 1000 µmol m^–2^ s^–1^ (Fig. 5). *W*_i_ at 1000 µmol m^–2^ s^–1^ can be grouped into six heavily overlapping statistical groups within these 10 cultivars, with IS23496 having the highest average *W*_i_ at 1000 µmol m^–2^ s^–1^ and IS36556 having the lowest. At 100 µmol m^–2^ s^–1^, there are four statistical groups (a–d), and IS16044 has the highest average *W*_i_; the lowest average *W*_i_ at 100 µmol m^–2^ s^–1^ was again observed in IS36556 and EC844904.

When assessing all 43 accessions (Supplementary Fig. S7A), *W*_i_ at 1000 µmol m^–2^ s^–1^ light exposure was also significantly greater than at 100 µmol m^–2^ s^–1^ (*P*<0.05) in all observed accessions except IS10978, a traditional cultivar from the USA (*P*=0.511). Eleven heavily overlapping statistical groups of *W*_i_ (a–k) at 100 µmol m^–2^ s^–1^ and 13 (a–m) at 1000 µmol m^–2^ s^–1^ were identified. The accession with the highest average *W*_i_ at 1000 µmol m^–2^ s^–1^ was IS40583 (BTx623), a research line from the USA, which had a statistically higher *W*_i_ than 30 other accessions. At 100 µmol m^–2^ s^–1^, however, while IS40583 still had a high *W*_i_, fitting into groups ‘a–d’, its *W*_i_ was only statistically greater than that of 16 other accessions. Meanwhile the accession with the lowest *W*_i_ at 1000 µmol m^–2^ s^–1^, the Nigerian landrace variety IS36556, was only significantly lower than 27 of the 43 tested accessions but the *W*_i_ of IS36556 at 100 m^–2^ s^–1^ was significantly lower than that of 36 of the 43 accessions. By directly comparing the *W*_i_ at 100 µmol m^–2^ s^–1^ with that at 1000 µmol m^–2^ s^–1^ for all measured accessions (Supplementary Fig. S7B), a significant correlation between these parameters was seen (*R*=0.49, *P*=0.00077), showing that accessions with higher *W*_i_ tended to have consistently greater efficiency at both high and low light than those with lower *W*_i_.

### Stomatal anatomy of sorghum varies by accession and by leaf surface

**Fig. 6.**
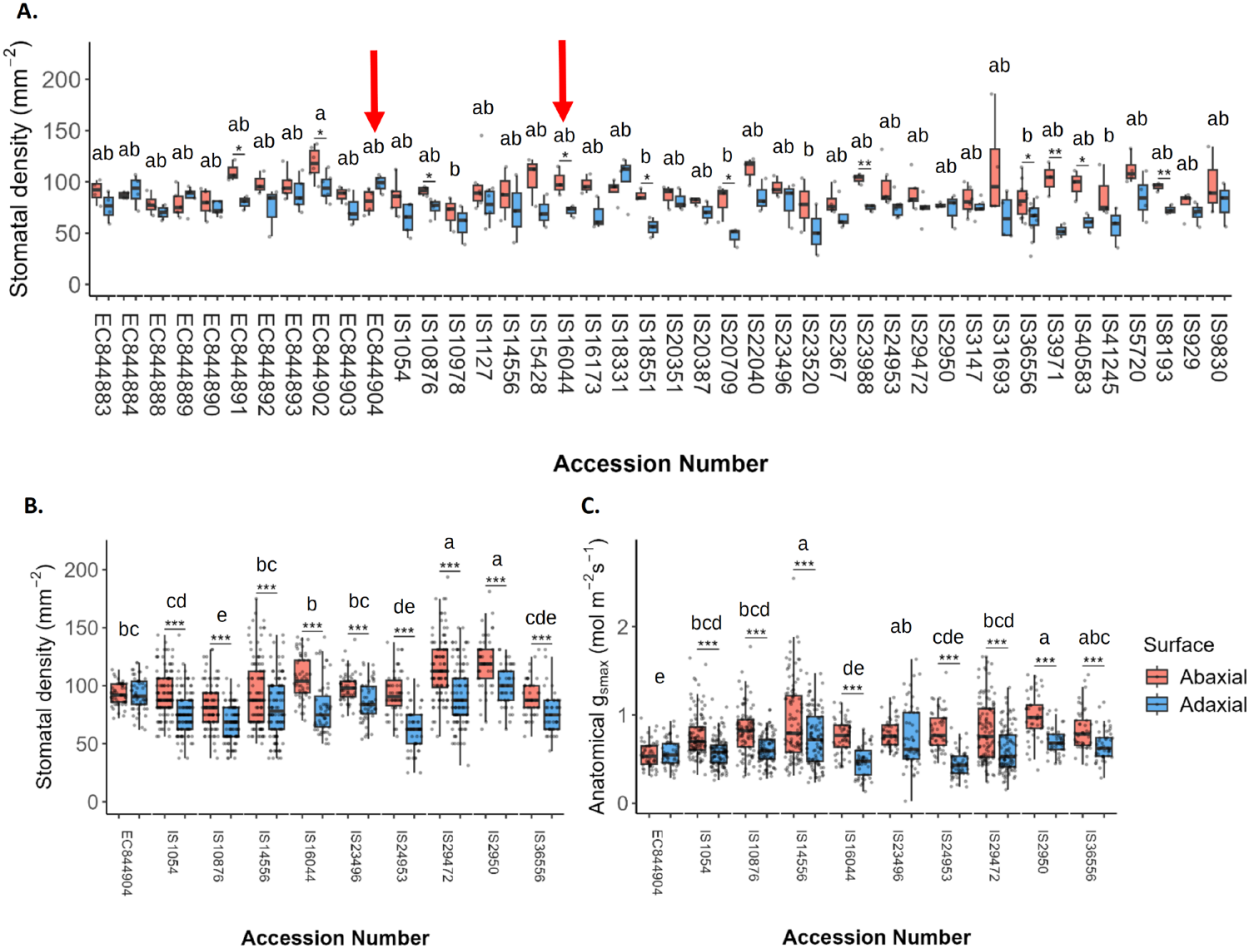
Variation of stomatal anatomy by leaf surface in sorghum. (A) Stomatal density of all accessions tested in this study measured from one field of view per leaf surface per plant for 2–6 plants per accession. Red arrows indicate accessions with the lowest *g*_smax_ (EC844904) and the second lowest (IS16044). (B) Stomatal density of 10 representative sorghum accessions measured from nine fields of view per leaf surface per plant for six plants per accession. (C) Maximum anatomical stomatal conductance (anatomical *g*_smax_) of 10 representative sorghum accessions calculated from the stomatal densities in (B) and the measured guard cell length and pore length of nine stomata per leaf surface per plant for six plants per accession. In each graph, separate measurements are shown for the abaxial (red) and adaxial (blue) leaf surface of each accession. Asterisks (*) above accessions indicate a significant difference between abaxial and adaxial leaf surfaces for the given accession calculated by ANOVA (***P< 0.0001, **P=0.001–0.01, *P=0.01–0.05). Letters indicate significantly different groupings of ANOVA results (*P*≤0.05) of accessions based on combined abaxial and adaxial values for each accession calculated using a Tukey’s test, *n*=6.

Stomatal density (SD) was measured on the abaxial (AB) and adaxial (AD) surface of all 43 sorghum accessions (Fig. 6A) and showed a range of SD values from 185 stomata mm^–2^ (IS31693) to 51 mm^–2^ (IS10978 and IS23520) for the AB surface and 121 mm^–2^ (IS18331) to 28 mm^–2^ (IS3583 and IS23520) for the AD surface. The accession with the highest average SD across both leaf surfaces was EC844902, an accession from Mali, with an average of 106.5 stomata mm^–2^, which was significantly higher than the four accessions with the lowest SD; IS3583 (65.0 mm^–2^), IS23520 (64.8 mm^–2^), IS10978 (64.6 mm^–2^), and IS20709 (64.1 mm^–2^). At this resolution of SD measurement, the average SDs of the remaining 38 accessions were statistically comparable with those of all 43 tested accessions.

SD was generally higher on the AB compared with all AD surface for all accessions; however, these differences were only significant (*P*<0.05) for 11 lines (Fig. 6A). In order to further analyse and validate these findings for the 10 selected lines, a more in-depth analysis was performed on stomatal anatomical features, including SD measured from nine fields of view per leaf surface per plant for six plants per accession (Fig. 6B) (where previously a single field of view had been measured per surface per plant for 2–6 plants per accession) and guard cell length and stomatal pore length, which were each measured from nine stomata per leaf surface per plant for six plants per accession. These data were used to calculate maximum anatomical stomatal conductance (anatomical *g*_smax_) for each leaf surface (Fig. 6C).

When comparing SD alone in these selected accessions (Fig. 6B), sorghum in nine of the 10 accessions again had significantly higher SD on the AB than on the AD leaf surface (*P*<0.0001). In this case, only EC844904 was observed to have no significant difference between the AB and AD leaf surface, with a statistically similar SD on each (*P*>0.05). Average SD across the combined leaf surfaces in Fig. 6B can be separated into five groups amongst these 10 plants. The highest stomatal densities are seen in the Lesothan landrace variety, IS29472, and the American breeding line IS2950, both of which are statistically comparable with each other and have a significantly greater SD than all other accessions. IS10876, a Nigerian landrace, has the lowest average SD of the observed accessions, with a statistically lower average SD than all but two of the other accessions. There was no relationship between stomatal size and density.

Anatomical *g*_smax_ showed that sorghum typically had a greater potential *g*_s_ on the AB leaf surface (Fig. 6C). Eight of the 10 selected lines presented significantly greater (*P*<0.0001) anatomical *g*_smax_ on the AB surface than on the AD surface. EC844904 and IS23496 showed no significantly different anatomical *g*_smax_ between surfaces (*P*>0.05). Of these 10 accessions, IS14556, an Ethiopian landrace variety, had significantly higher anatomical *g*_smax_ over the combined leaf surfaces than all but three other varieties, IS36556, a Nigerian landrace, IS23496, an Ethiopian landrace, and IS2950, a research line from the USA. Pooling the SD data for all of the 43 accessions revealed a strong correlation between AD and AB SD (Supplementary Fig. S8; *R*=0.22, *P*=0.048), showing that the AB SD was typically higher than the AD SD, and an increase in SD on one surface was typically accompanied by an increase on the opposite surface. Leaf anatomical parameters such as SD and guard cell length and pore size were significantly correlated (*P*<0.05) to the *g*_s_ and *A* values reached at the end of a photosynthesis induction (Supplementary Fig. S8). However, these correlations had relatively low *R*^2^, with values ranging from 0.12 to 0.2.

## Discussion

As expected, sorghum can operate at low *g*_s_, whilst maintaining relatively high photosynthesis levels compared with C_3_ plants. The resulting low operating *C*_i_ was in a relatively small range of values among accessions and near the edge of what is required for photosynthetic saturation, with values around or even sometimes below the 100–150 µmol mol^–1^ range reported previously (Pearcy and Ehleringer, 1984; Ehleringer and Monson, 1993). Despite similar *C*_i_ values, a large diversity in *g*_s_ and *A* was observed in the 43 sorghum accessions studied here, and an extremely strong correlation and synchronicity between *A* and *g*_s_ at different light levels was clearly evident. Our results highlight a tight control of gaseous exchange in both dynamic and steady-state conditions in sorghum, with implications for water use efficiency. Accessions with the greatest *A* displayed the highest *g*_s_ values, and vice versa, in both steady and non-steady-state conditions (Figs 1–3). These findings provide new insights into the coordination of *A* and *g*_s_, and potential novel targets for manipulation to increase crop production.

Whilst significant diversity in photosynthetic capacity including the maximum quantum yield (Fig. 1C) and light-saturated rate of photosynthesis (*A*_sat_) (Fig. 1D) was observed, what was particularly interesting and surprising was the near identical patterns of behaviour in *g*_s_ in response to changing irradiance as those recorded for *A* (Fig. 1A, B). Light response curves are most often utilized to determine differences in photosynthetic performance, and measurements are carried out rapidly, with *A* typically recorded ~1–2 min after a change in light intensity in order to ensure that stomata remain open and do not influence the measurements (Parsons *et al.*, 1998). In the 10 accessions selected, the response of *A* and *g*_s_ was almost identical, with rapid adjustment in stomatal aperture, within 2 min of changing irradiance, resulting in a highly significant and tight correlation between *A* and *g*_s_ (Fig. 2) in all accessions (Supplementary Fig. S2). It is well established that there is a close correlation between *A* and *g*_s_ (Wong *et al.*, 1979) [which forms the basis of the Ball, Woodraw, Berry model (Ball *et al.*, 1987)] and that such synchronous behaviour optimizes carbon gain to water loss (Buckley, 2017). However, although these relationships are often conserved, they are not constant and under non-steady-state conditions significant deviation can be seen, often due to relatively slow *g*_s_ responses relative to *A* (Lawson *et al.*, 1998, 2010). Here we have demonstrated near linear relationships between *A* and *g*_s_ in response to dynamic changes in light intensity (Fig. 2) in all accessions, although variation in the absolute values was apparent. This was enabled by the rapid stomatal responses reported here (Fig. 4), which also helped maintain a constant *W*_i_ (Supplementary Fig. S6) under changing irradiance. Here we demonstrated an extremely tight (*R*^2^=0.99) coupling of *g*_ssat_ and *A*_sat_ (Fig. 3), along with a maintenance of *C*_i_, illustrating an impressive control of gaseous fluxes via stomata that are linked directly to mesophyll CO_2_ demands, enabling sorghum to maintain high steady-state *W*_i_ without compromise to *A*.

The observed diversity of *g*_s_ and *A* values whilst maintaining a tight relationship suggests that there is a common signal to which stomata respond rapidly, which facilitates high water use efficiency. Such a strong relationship between *g*_s_ and *A* has often been interpreted as a diffusional constraint on photosynthesis; however, it could also result from a strong signal regulating *g*_s_ to maintain saturating *C*_i_ while limiting water loss. Stomatal conductance in C_4_ plants is usually not limiting in non-stressed plants as long as operational *C*_i_ is allowed to reach saturation (Bunce, 2005; Ghannoum, 2008). The assumption of a signal coordinating *A* and *g*_s_ is supported by the relatively stable and expected *C*_i_:*C*_a_ ratios of between 0.3 and 0.4 (Jones, 1983) (Supplementary Fig. S1) and stable *C*_i_ values (Supplementary Fig. S3) although these were a little lower than the expected ~100–150 µmol mol^–1^ in some accessions (Pearcy and Ehleringer, 1984; Ehleringer and Monson, 1993).

A number of studies have previously suggested that temporal responses to increasing light intensity lead to a disconnect between *A* and *g*_s_ (Lawson and Blatt, 2014; Kaiser *et al.*, 2016; McAusland *et al.*, 2016; Matthews *et al.*, 2017, 2018; Vialet-Chabrand *et al.*, 2017a, b; De Souza *et al.*, 2020; Yamori *et al.*, 2020) due to the difference in rapidity of these responses for *g*_s_ compared with *A* (McAusland *et al.*, 2016; Qu *et al.*, 2016; Matthews *et al.*, 2017). In C_3_ crops, stomatal responses are in general an order of magnitude slower than photosynthetic responses, resulting in diffusive limitation during stomatal opening or unnecessary water loss during stomatal closure (Lawson and Blatt, 2014; McAusland *et al.*, 2016; Faralli *et al.*, 2019b; De Souza *et al.*, 2020). In contrast, our observations displayed significant variation in the response of both *A* and *g*_s_, with dynamic responses that were atypically strongly coordinated in the short term (Fig. 4A, B). The speeds of *A* induction (τ_ai_) and the rapidity of increase in *g*_s_ (τ_i_) were <10 min for the vast majority of the lines (Fig. 4D). Although several C_3_ cereal crop species have been reported to have rapid stomatal responses, only rice, barley, and Miscanthus have been reported to be this fast in *g*_s_ kinetics (see McAusland *et al.*, 2016). In general stomatal opening and closing have been reported to be more rapid in grasses (Franks and Farqhuar 2007; Nunes *et al.*, 2022), which is thought to be due to the relationship between guard and subsidiary cells (Franks and Farqhuar, 2007; Raissig *et al.*, 2017). However, photosynthetic type also plays a role, with C_4_ plants reported to be more rapid than C_3_ plants (McAusland *et al*., 2016). Ozeki *et al.* (2022) also demonstrated that rapid stomatal closure contributed to higher water use efficiency in C_4_ compared with C_3_*Poaceae* crops. The speed of *A* induction was typically more rapid than for most field crops (McAusland *et al.*, 2016), fruit crops (Zhang *et al.*, 2022), as well as the model Arabidopsis (Burgess *et al.*, 2023). This suggests that sorghum has a mechanism that maintains both the coordination between steady-state *A* and g_s_ and the extremely tight synchrony of their responses through time. In C_3_ crops, different hypotheses have been proposed (Lawson *et al.*, 2014, 2018); there is strong support for a positive feedback loop that controls stomatal aperture based on mesophyll photosynthesis, with *C*_i_ potentially coordinating the response (Farquhar *et al.*, 1978; Vialet-Chabrand *et al.*, 2021). A coordination based on *C*_i_ would result in a relatively constant *W*_i_ as observed in this study. This would suggest an extreme sensitivity of sorghum stomata to internal CO_2_ concentration that remains to be tested.

The generally fast stomatal responses observed here lead to a strong correlation between the speed of *g*_s_ and the speed of *A*, confirming the importance of the rapidity of stomatal responses in photosynthetic induction (Supplementary Fig. S4) (Long *et al.*, 2022). As stomatal conductance is determined by both anatomical and biochemical features (Hetherington and Woodward, 2003; Franks and Farquhar, 2007), we measured SD on both the AD and AB leaf surface (Fig. 6). As expected, a greater number of stomata were generally observed on the AD surface; however, these differences were not always significant, with 17 of the 43 species having no significant difference in SD between AB and AD surfaces (Fig. 6). Generally, AD and AB SD were correlated (Supplementary Fig. S8), and similar patterns have been observed in wheat, suggesting a common signal that coordinates stomatal anatomy between the two surfaces (Wall *et al.*, 2022), although to date the signal transduction pathway has not been fully elucidated (Santos *et al.*, 2021). Differences in SD between surfaces are well established in amphistomatous leaves (Ticha, 1982), although the impact on functional responses is less well understood (Mott *et al.*, 1982; Mott and O’Leary, 1984; Xiong and Flexas, 2020; Santos *et al.*, 2021; Wall *et al.*, 2022). Anatomical *g*_smax_ provides a measure of the maximum potential *g*_s_ and therefore is indicative of possible functional differences (Lawson *et al.*, 1998; Franks and Farquhar, 2001; Lawson and Morison, 2004; Dow and Bergmann, 2014). All accessions had a low *g*_smax_ compared with C_3_ crops, which is in accordance with previous results (Taylor *et al.*, 2012). EC844904 had the lowest leaf *g*_smax_, with IS16044 the second lowest; interestingly, these two accessions also exhibited the lowest and one of the highest leaf *W*_i_ values (Fig. 5), illustrating that *W*_i_ is strongly influenced by stomatal behaviour. As no correlation was observed between the speed of *g*_s_ responses and SD, this suggests that this behaviour was probably not driven by variation in anatomy but supports the idea that stomatal biochemistry and metabolism contribute more to the rapidity of *g*_s_ (Lawson and Blatt, 2014). Surprisingly, none of the parameters measured in this study appears to be strongly linked to the country of origin of the accessions, suggesting that development of variability between these accessions was not strongly driven by geographic location.

In conclusion, this study has demonstrated that sorghum displays one of the most rapid stomatal responses in a crop species, with the majority of accessions responding within ≤5 min, and validates the suggestions that stomatal rapidity is greater in C_4_ than in C_3_ plants (McAusland *et al.*, 2016; Nunes *et al.*, 2022; Ozeki *et al.*, 2022). The strong coordination of *A* and *g*_s_ under both steady-state and dynamic conditions with a diversity in absolute values has not previously been reported and implies that a mesophyll signal or *C*_i_ tightly regulates stomatal conductance in order to minimize water loss and maximize photosynthesis. Differences in stomatal kinetics and levels reached at each light intensity were not driven by variation in leaf anatomy, but due to functional differences that determine osmoregulation, such as guard cell metabolism or ion channel sensitivity (Blatt and Clint, 1989; Ache *et al.*, 2000; Hosy *et al.*, 2003; Lawson and Blatt, 2014; Lawson *et al.*, 2014; Jezek and Blatt, 2017). Therefore, sorghum represents a prime candidate for uncovering the elusive mechanisms that coordinate *A* and *g*_s_, as well as guard cell traits that permit such rapid *g*_s_ responses and using such information to design crops with high *A* without incurring significant water losses and eroding *W*_i_. Ideally, it is advantageous to have crops that have rapid *g*_s_ responses, that are closely coupled with mesophyll demands for CO_2_ to maximize *A*, water use, and overall crop productivity. The mechanisms that enable sorghum to achieve this could provide as yet unknown targets to improve productivity in both C_3_ and C_4_ crops.

## Supplementary data

The following supplementary data are available at *JXB* online.

Table S1. *Sorghum bicolor* L. Moench accessions used in this study.

Fig. S1. Light response curves and relationship between maximum apparent quantum yield and photosynthetic CO_2_ assimilation for the 43 sorghum accessions.

Fig. S2. Rate of change of CO_2_ assimilation rate (*A*) relative to stomatal conductance to water (*g*_s_) in response to changes in PPFD.

Fig. S3. Temporal responses of internal CO_2_ concentration (*C*_i_) to increase in PPFD.

Fig. S4. Time constants of carbon assimilation and stomatal opening.

Fig. S5. Association between the rate of induction of carbon assimilation and speed of stomatal opening.

Fig. S6. Temporal response of intrinsic water use efficiency (*W*_i_) to a rapid increase in PPFD irradiance.

Fig. S7. Changes in intrinsic water use efficiency (*W*_i_).

Fig. S8. Matrix representing multiple regressions between modelled parameters describing the rapidity of stomatal and net CO_2_ assimilation response and leaf anatomical parameters.

erae389_suppl_Supplementary_Materials

## Data Availability

Data are available on request from the corresponding author.
