# Supplementary material for: Fast stomatal kinetics in sorghum enable tight coordination with photosynthetic responses to dynamic light intensity and safeguard high water use efficiency
Source: J Exp Bot. 2024 Sep 18;75(21):6796–809. doi: 10.1093/jxb/erae389 (PMC11565209; doi:10.1093/jxb/erae389)
Supplement: erae389_suppl_Supplementary_Materials [file erae389_suppl_supplementary_materials.pdf]

Supplemental Table S1. *Sorghum bicolor* L. Moench Accessions used in this study

Accession Number	Alternative Name	Country of Origin	Biological Status
IS 929	PI 568989	Sudan	Traditional cultivar/Landrace
IS 1054	PI 248264	India	Advanced/Improved cultivar
IS 1127	A 35	India	Traditional cultivar/Landrace
IS 2367	Q2-4-74	Nigeria	Traditional cultivar/Landrace
IS 2950	R-16 / SA 7526-1-20-8-2-10-1	USA	Breeding/Research material
IS 3147	DL 60/114	South Africa	Traditional cultivar/Landrace
IS 3583	HSD 7867	Sudan	Traditional cultivar/Landrace
IS 3971	IC 8946	India	Traditional cultivar/Landrace
IS 5720	Janera pipla	India	Traditional cultivar/Landrace
IS 8193	KA 4	Uganda	Breeding/Research material
IS 9830	PI 563310	Sudan	Traditional cultivar/Landrace
IS 10876	BO50	Nigeria	Traditional cultivar/Landrace
IS 10978		USA	Traditional cultivar/Landrace
IS 14556		Ethiopia	Traditional cultivar/Landrace
IS 15428	2-1-6-7	Cameroon	Traditional cultivar/Landrace
IS 16044	4-3-26-3	Cameroon	Traditional cultivar/Landrace
IS 16173	4-1-3-15	Cameroon	Traditional cultivar/Landrace
IS 18331	N 13	India	Breeding/Research material
IS 18551	Jijwerjere 935	Ethiopia	Breeding/Research material
IS 20351	SG 2303	Niger	Traditional cultivar/Landrace
IS 20387	SG 2375	Niger	Traditional cultivar/Landrace
IS 20709	HDW 128	USA	Traditional cultivar/Landrace
IS 22040	Yerra jonna	India	Traditional cultivar/Landrace
IS 23496		Ethiopia	Traditional cultivar/Landrace
IS 23520	Sima	Ethiopia	Advanced/Improved cultivar
IS 23988	AR 220	Yemen	Traditional cultivar/Landrace
IS 24953	No. 2360	Zambia	Traditional cultivar/Landrace
IS 29472		Lesotho	Traditional cultivar/Landrace
IS 31693	S. bicolor 71	Algeria	Traditional cultivar/Landrace
IS 36556	S-35	Nigeria	Traditional cultivar/Landrace
IS 40583	BTX 623	USA	Breeding/Research material
IS 41245	296-B	India	Breeding/Research material
EC 844883	Framida	Birkina Faso	Breeding/Research material
EC 844884	Grinkan / 02-sb-f4dt-275	Mali	Breeding/Research material
EC 844888	DOUA-G	Ivory Coast	Breeding/Research material
EC 844889	GNOSSICONI	Birkina Faso	Breeding/Research material
EC 844890	RIBDAHU	Niger	Breeding/Research material
EC 844891	SAMBALMA	Nigeria	Breeding/Research material
EC 844892	GPN01 S01 -9-3-3-VAR	Mali	Breeding/Research material
EC 844893	GPN01 S01 -9-3-1-1	Mali	Breeding/Research material
EC 844902	GPN01 S01 -9-V1-2-34- 2	Mali	Breeding/Research material
EC 844903	CSM63-E	Mali	Breeding/Research material
EC 844904	BBISS-08	Wild (unknown region)	Breeding/Research material

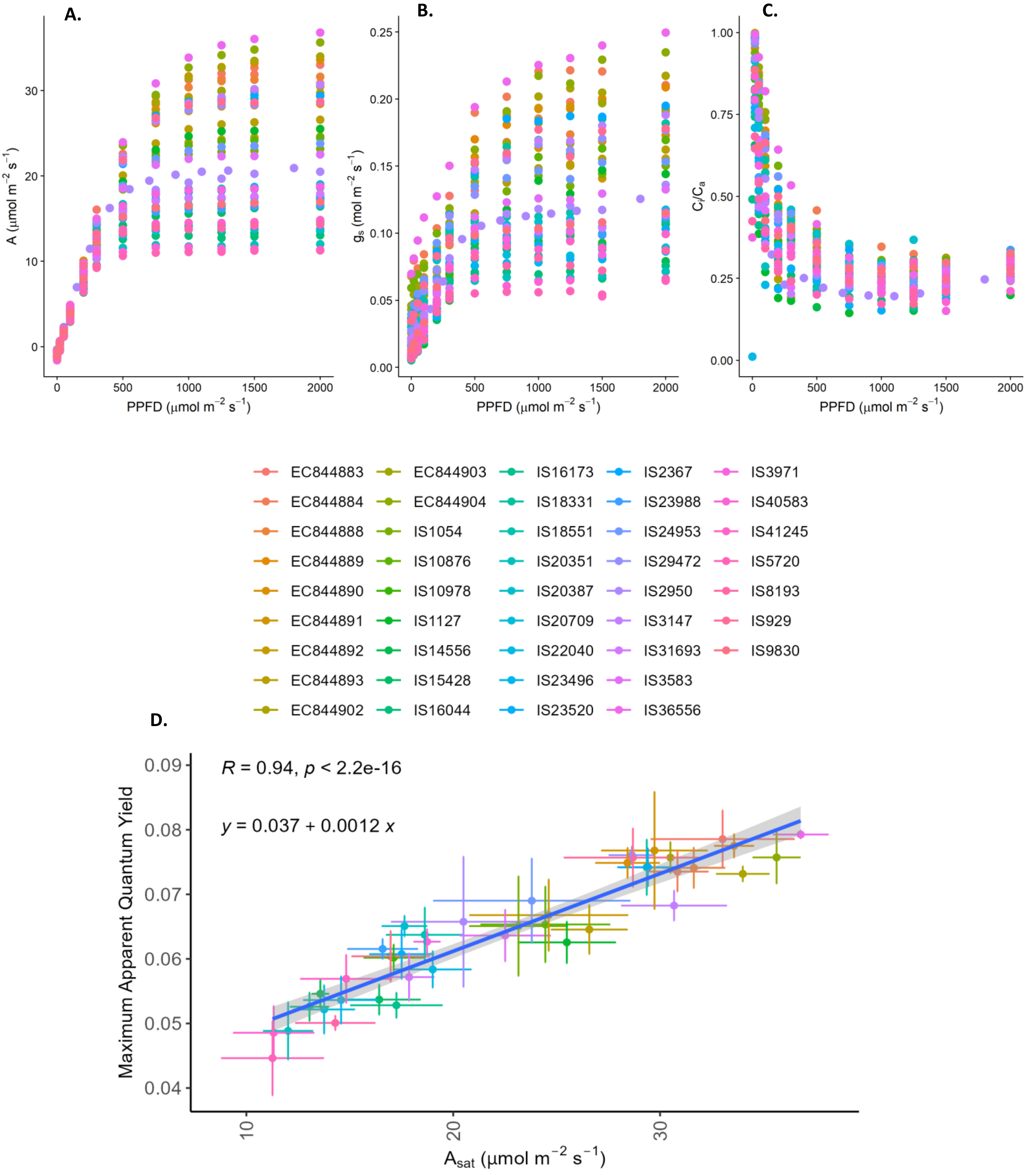

Supplementary Fig. S1. Relationship between maximum apparent quantum yield and photosynthetic CO2 assimilation at 2000 µmol m-2 s-1 (Asat) for the 43 sorghum accessions tested in this study. Points indicate mean values for the accession of the associated colour. Error bars indicate standard error of the mean, n= 3-8. Line represents R= 0.94, p <2.2e-16 with a regression formula of y = 0.037 + 0.0012 x.

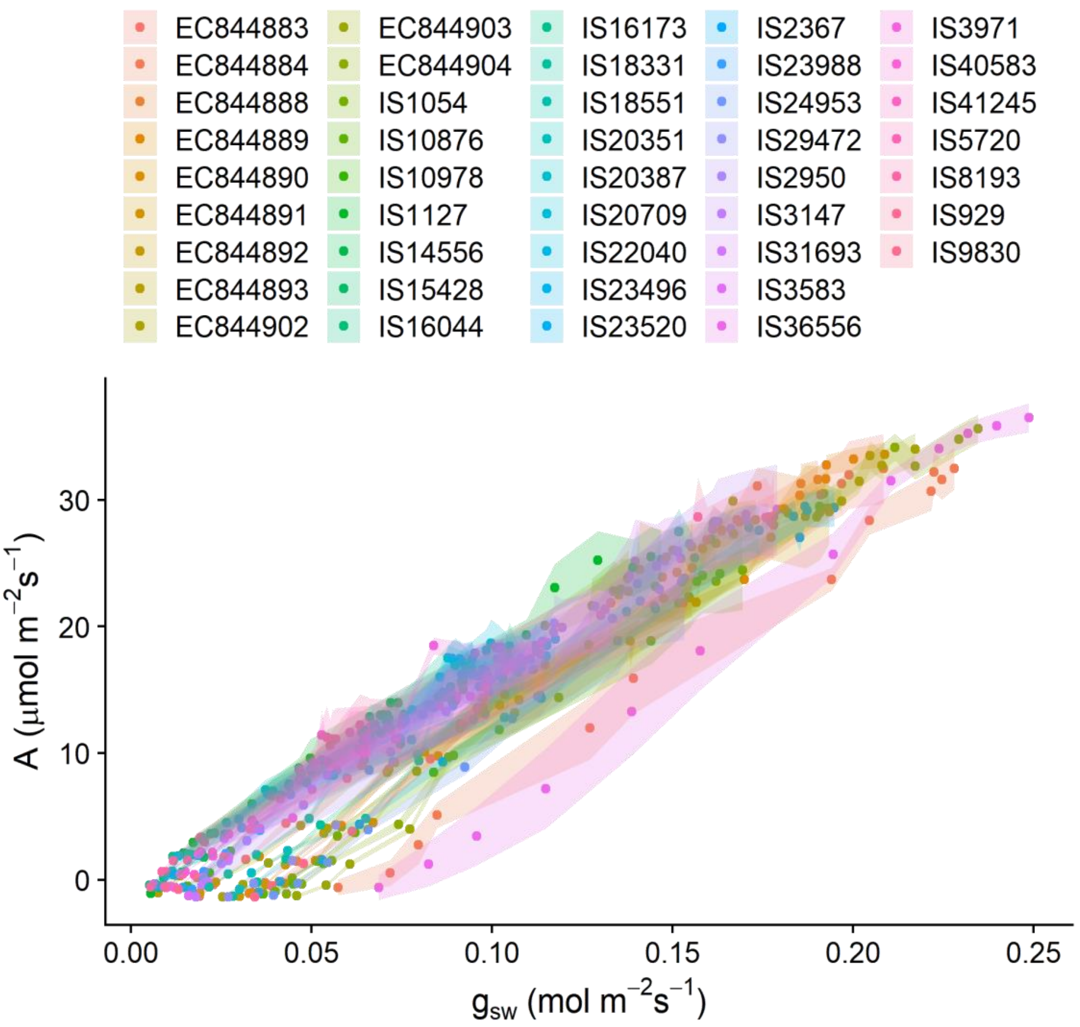

Supplementary Fig. S2. (A.) Rate of change of CO2 assimilation rate (A) relative to stomatal conductance to water (gs) in response to changes in PPFD irradiance intensities from 0 to 2000 μmol m-2 s-1, as shown in Fig. 2. Data shown here for all 43 tested accessions. Points indicate mean average of 3-6 plants and ribbons indicate standard error. (B.) Light saturated rate of photosynthetic CO2 assimilation (Asat; A at 2000 μmol m-2 s-1) relative to stomatal conductance under saturating light (gssat; gs at 2000 μmol m-2 s-1) for each tested sorghum accession. Points indicate mean average values for accessions as indicated by colour. Error bars indicate standard error of the mean, n= 3-8. Shaded ribbon represent standard error around the mean. Line represents R = 0.99, p <2.2e-16 with a regression formula of y = 1.3 + 150 x.

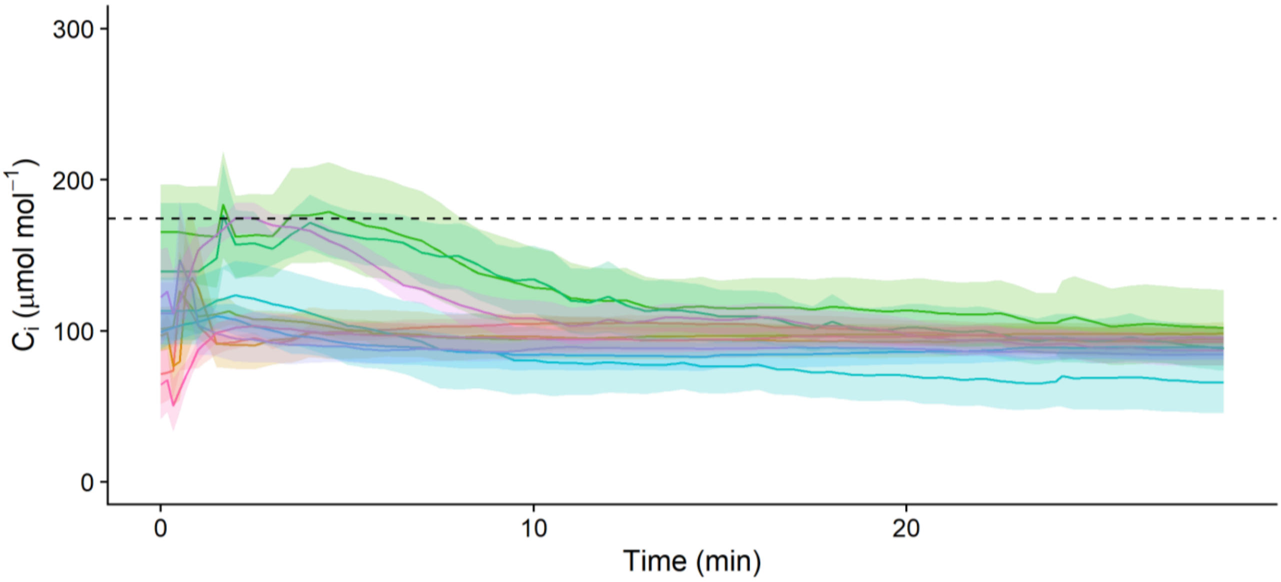

Supplementary Fig. S3. Temporal responses of internal CO2 concentration (Ci) to a rapid increase in PPFD irradiance from 100 µmol m-2 s-1 to 1000 µmol m-2 s-1 in the ten representative sorghum accessions. n= 4-8 Shaded ribbon represent standard error around the mean

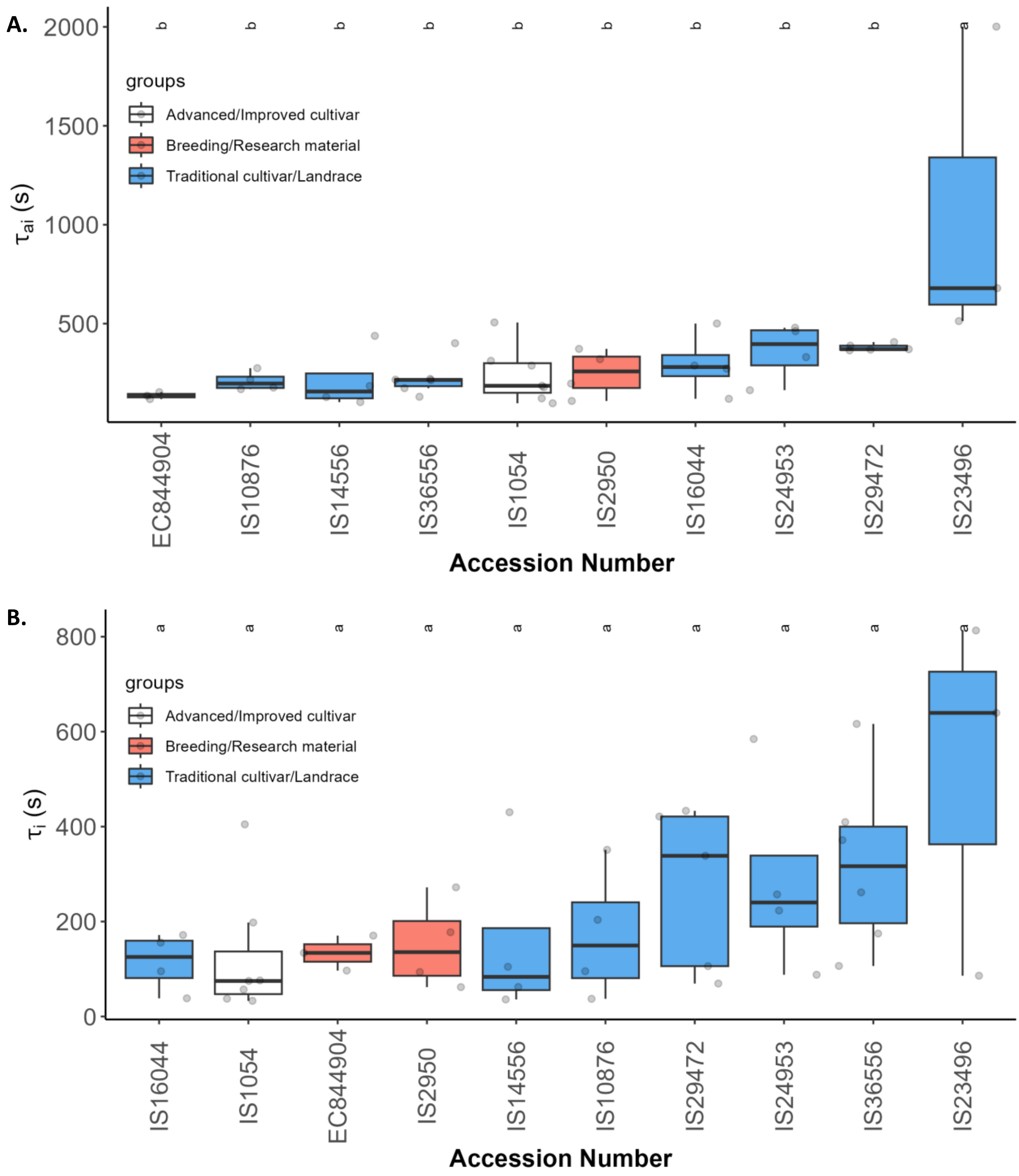

Supplementary Fig. S4. Time constants of (A.) light saturated rate of carbon assimilation (τai) and (B.) stomatal opening (τi) in seconds, shown here for 10 representative sorghum accessions. Accession numbers in A. and B. ordered from lowest to highest mean τai. Letters indicate significantly different groupings of ANOVA results ($p \leq 0.05$) calculated using a Tukey's test, n= 3-8.

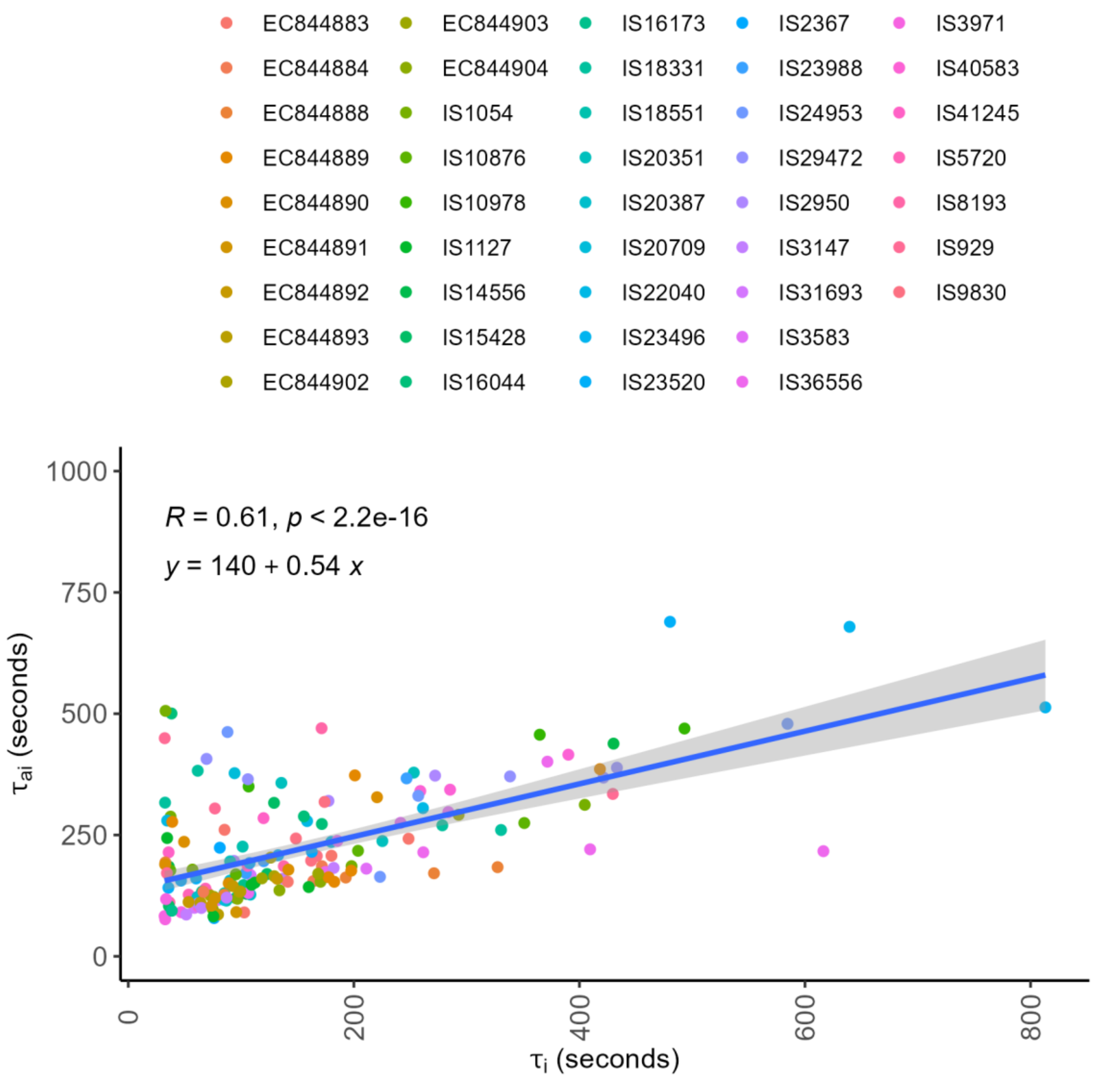

Supplementary Fig. S5. Association between the light saturated rate of carbon assimilation (τai) and speed of stomatal opening (τi) in seconds. Each point indicates an individual plant. Accession number is indicated by colour. Line represents R = 0.67, p <2.2e-16 with a regression formula of y = 150 + 0.51 x, n= 3-8.

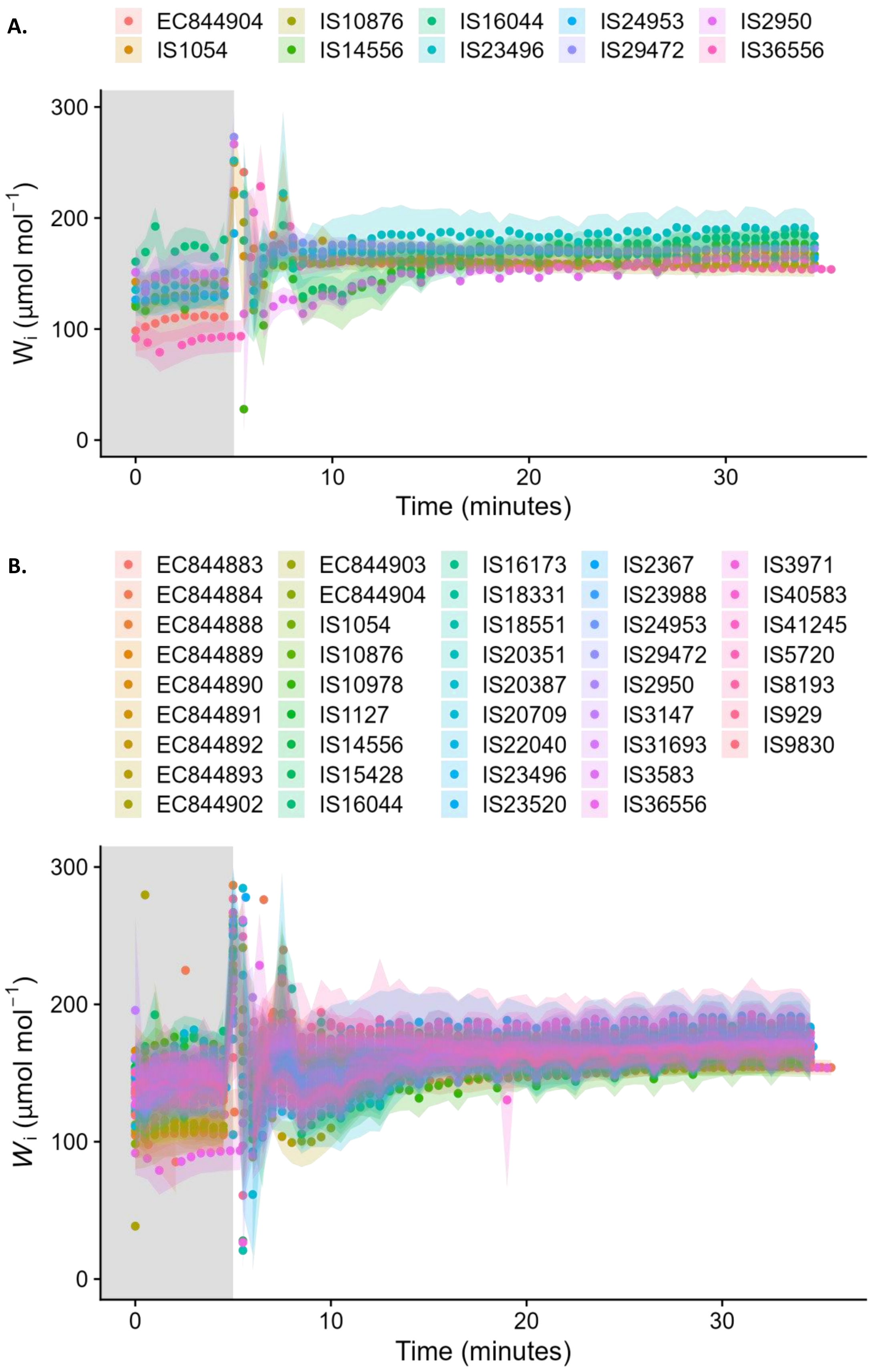

Supplementary Fig. S6. Temporal response of intrinsic water use efficiency (Wi) to a rapid increase in PPFD irradiance from 100 µmol m-2 s-1 (shaded area) to 1000 µmol m-2 s-1 in (A.) ten representative sorghum accessions and (B.) all measured accessions. n= 3-8. Shaded ribbon represent standard error around the mean.

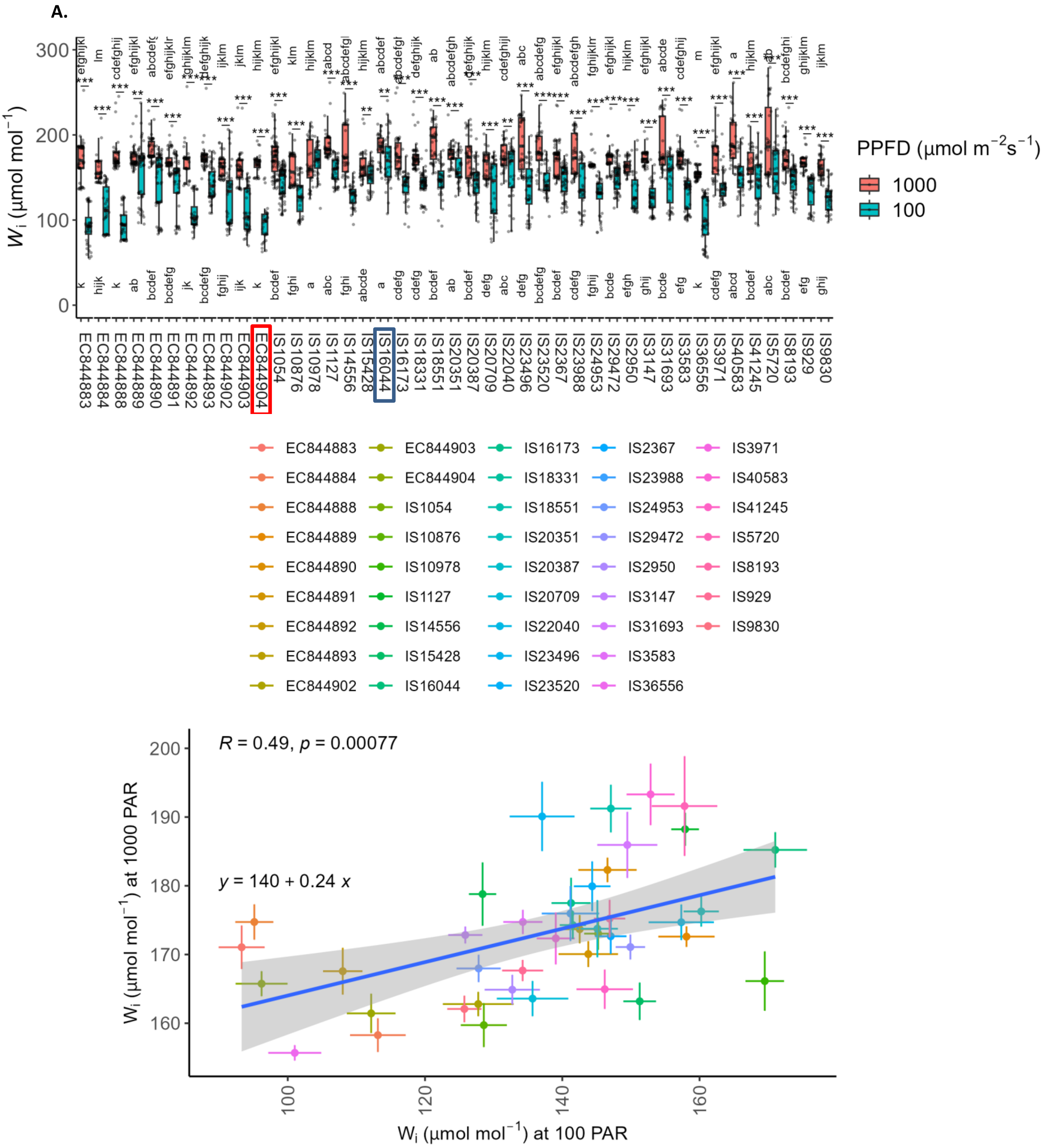

Fig. S7. (A) Changes in intrinsic water use efficiency (Wi) in response to photosynthetic photon flux density (PPFD). Average Wi measured every 30 seconds for 5 min in dark adapted plants exposed to 100 µmol m-2 s-1 of light (blue boxplots) compared to average Wi measured every 30 seconds for 5 min after 30 min of exposure to 1000 µmol m-2 s-1 of PPFD irradiance (red boxplots). Graph shows data from 43 sorghum accessions. Asterisks (*) above accessions indicate a significant difference between 100 µmol m-2 s-1 and 1000 µmol m-2 s-1 treated plants for the given accession calculated by ANOVA (*** = p<0.0001, ** = p 0.001–0.01, * = p 0.01–0.05). Letters indicate significantly different groupings of ANOVA results (p ≤ 0.05) of accessions for plants exposed to 100 µmol m-2 s-1 (letters below boxplots) or 1000 µmol m-2 s-1 (letters above boxplots) of PPFD irradiance. Groupings were calculated using a post-hoc Tukey's test. (B) Relationship between Wi at 100 and 1000 µmol m-2 s-1 for the 43 sorghum accessions. Points indicate mean values for the accession of the associated colour. Error bars indicate standard error of the mean, n= 2-6. Line represents R = 0.49, p = 0.00077 with a regression formula of y = 140 + 0.24 x.

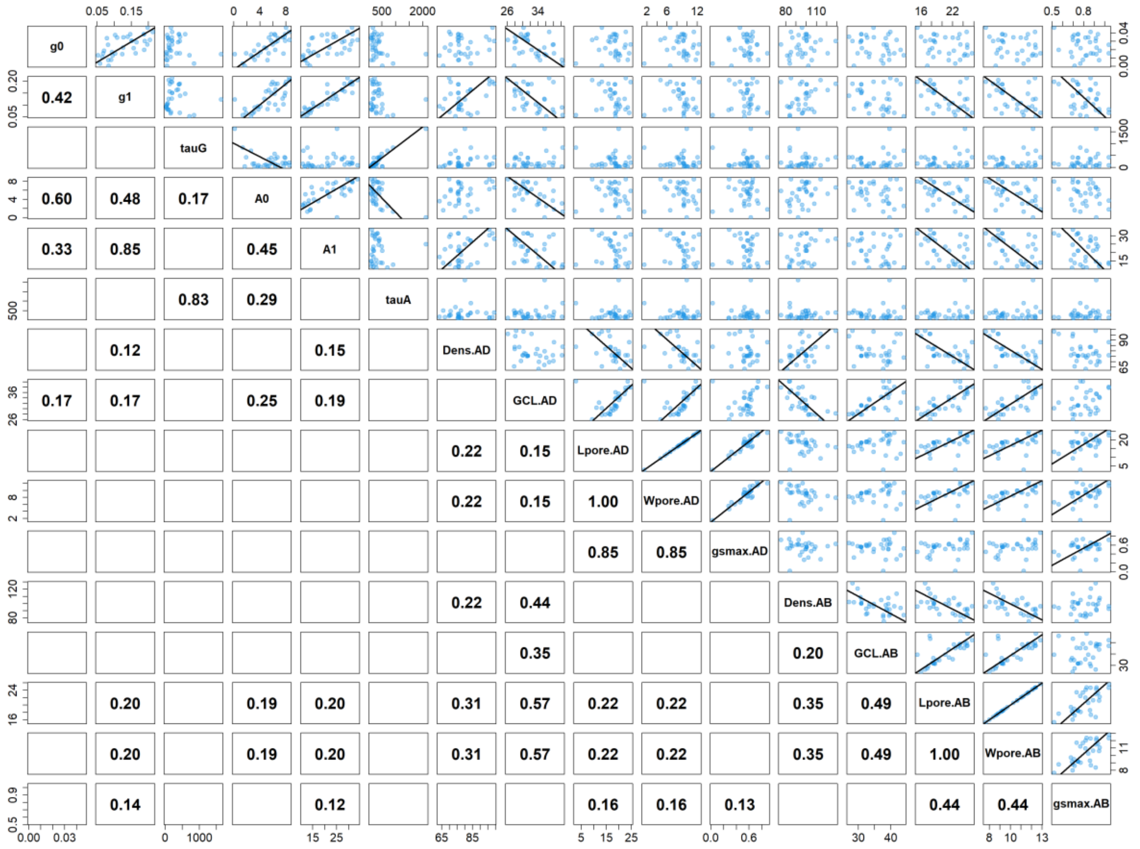

Supplementary Fig. S8. Matrix representing multiple regressions (using standard major axis) between modelled parameters describing the rapidity of stomatal and net CO2 assimilation response and leaf anatomical parameters. Values in the lower panel represent the R2 of the regression for each parameter pair, n= 3-8. Only significant regressions (p <0.05) were represented by a line in the upper panel. Parameters g0, A0 and g1, A1 represent the initial and final value for gs and A during a step change in light intensity; τg and τa represent the time constant for an increase in gs and A; Dens represents the stomatal density; GCL represents the guard cell length; Lpore and Wpore represent the length and width of the stomatal pore; gsmax represents the maximum anatomical gs; Suffixes AD and AB represent the adaxial and abaxial side of the leaf.
